# Management of rare side effects of peginterferon and ribavirin therapy during hepatitis C treatment: a case report

**DOI:** 10.4076/1757-1626-2-7429

**Published:** 2009-06-12

**Authors:** Kamal EL-Atrebi, Hala T El-Bassyouni

**Affiliations:** 1 General Medicine and Hepatology Department, National Hepatology and Tropical Medicine Research InstituteEgypt; 2 Clinical Genetics Department, National Research CentreEgypt

## Abstract

Cardiac arrhythmias and syncope are rare consequence of therapy with pegylated interferon. We report a 55-year-old Egyptian woman who developed palpitation and syncopal attacks twice within 3 months after starting therapy with pegylated interferon α2b and ribavirin for the treatment of chronic hepatitis C virus. Hepatitis C virus-RNA was undetectable after 12 week. The electrocardiogram holter revealed ventricular extra-systoles on top of sinus tachycardia. pegylated interferon α2b therapy was continued but with a reduced dose. Close follow up revealed neither palpitation nor syncope and hepatitis C virus-RNA was undetectable at 24 and 48 weeks of treatment. In conclusion, our study suggests readjustment of the dose of pegylated interferon α2b therapy to avoid these serious side effects.

## Introduction

Chronic hepatitis C affects 300 million people worldwide [1]. Treatment of hepatitis C virus (HCV) with interferon alpha 2b (IFN) and ribavirin therapy has been shown to be effective, with 41-47% of patients achieving a sustained virologic response [2]. A regimen of peginterferon α2b (peg-IFN) and ribavirin has proven to be more effective than a regimen of conventional interferon and ribavirin with overall sustained virologic response rates 54-56% [3-5].

In spite of the current popularity of peg-IFN α2b and ribavirin therapy, many side effects have been reported [6]. Flu-like symptoms such as fever, chills, muscle ache; nausea, vomiting, and fatigue are common side effects of treatment. Depression and related symptoms, such as anxiety, irritability, insomnia, and mental confusion, are not rare and may be significant in patients with a previous history. Common hematologic side effects that require monitoring include neutropenia and thrombocytopenia. Withdrawal rates in IFN-based combination studies due to side effects have ranged from 6% to 7% [7].

In addition, Peg-IFN therapy has been reported to affect cardio-vascular system (cardiac arrhythmia) as reported in product information. One study reported that un-explained lipothymia (syncope or sudden fainting) resulted in discontinuation of the treatment [8].

## Case presentation

A 55-years-old Egyptian woman with chronic hepatitis C whose weight was 99 kg was treated with Peg-IFN-α2b at a dose of 1.5 μg/kg per week subcutaneously (150 μg/week) and ribavirin 1200 mg/day. Her drug and past medical history was unremarkable, she was not hypertensive nor had cardiac abnormalities. Her basic resting ECG was normal. Her hemoglobin (Hb) was 14.9 g/dl. Her follow up on week 1, 2 and 4 showed no problems except a reasonable progressive decrease of her Hb to 12.5. On week 8, she complained of dyspnea grade II and dizzy spills; her Hb was 11.1 g/dl. The ribavirin was decreased to 1000 mg/day. On week 12, her HCV-RNA level estimated by sensitive polymerase chain reaction (PCR)-based assay was negative, but unfortunately, she complained of two attacks of palpation followed by syncopal attacks. An ECG holter to monitor her cardiac arrhythmia and echocardiography and testing her electrolytes were done. ECG holter revealed ventricular ectopic activity in the form of unifocal ventricular extrasystoles (VES) and sinus tachycardia (Max HR was 156 beats/min).

Echocardiography was normal. Electrolytes and thyroid profile were within normal range. Finally, the medication was continued but it was decreased to 120 μg/week. Follow up did not show any more palpitation, syncopal attacks or other side effects. Treatment was continued for 48 weeks continuously. The HCV RNA on week 24 and 48 was undetectable.

## Discussion

Side effects of IFN and ribavirin combination therapy for HCV have been documented; however, cardiac arrhythmia and syncopal attacks are rare reported side effects [9].

Cardiac arrhythmia is reported only in the Peg interferon product information and one big study conducted on 441 chronic HCV patients reported that unexplained syncopal attacks occurred in 11 patients resulting in discontinuation of the treatment [8].

Therefore, any syncopal attacks should be fully investigated before discontinuing the medication and the risk - benefit should be explored.

## Conclusion

In conclusion, dose readjustment instead of discontinuation of IFN can be beneficial in dealing with these serious side effects without changing the viral response rate. However, further research is needed to delineate optimal therapeutic strategies in larger studies.

## Patient's perspective

I was infected with chronic hepatitis C. Then after starting my treatment, I suffered from irregular heartbeats followed by fainting. However, after decreasing the dose, the symptoms were alleviated.

## Abbreviations

None

## References

[bib-001] Patrick DM, Buxton JA, Bigham M, Mathias RG (2000). Public health and hepatitis C. Can J Public Health.

[bib-002] McHutchison JG, Manns M, Patel K, Poynard T, Lindsay KL, Trepo C, Dienstag J, Lee WM, Jak C, Garaud JJ, Albrecht JK (2002). International Hepatitis Interventional Therapy Group. Adherence to combination therapy enhances sustained response in genotype-1-infected patients with chronic hepatitis C. Gastroenterology.

[bib-003] Fried MW, Schiffman ML, Reddy KR, Smith C, Marinos G, Goncales FL, Haussinger D, Diago M, Carosi G, Dhumeaux D, Craxi A, Lin A, Hoffman J, Yu J (2002). Peginterferon alfa-2a plus ribavirin for chronic hepatitis C virus infection. N Engl J Med.

[bib-004] Hadziyannis SJ, Sette H, Morgan TR, Balan V, Diago M, Marcellin P, Ramadori G, Bodenheimer H, Bernstein D, Rizzetto M, Zeuzem S, Pockros PJ, Lin A, Ackrill AM (2004). PEGASYS International Study Group. Ann Intern Med.

[bib-005] Kang MJ, Jung EU, Park SW, Choi P, Kim JH, Park SJ, Park ET, Lee YJ, Lee SH, Seol SY (2008). Effects of pegylated interferon and ribavirin in Korean patients with chronic hepatitis C virus infection. Korean J Hepatol.

[bib-006] Martin P, Jensen D (2008). Ribavirin in the Treatment of Chronic Hepatitis C. J Gastroenterol Hepatol.

[bib-007] Reichard O, Norkrans G, Fryden A, Braconier JH, Sonnerborg A, Weiland O (1998). Randomized, double-blind, placebo-controlled trial of interferon alpha-2-b with and without ribavirin for chronic hepatitis C. Lancet.

[bib-008] http://www.hcvadvocate.org/news/review_archive_2002.asp.

[bib-009] Okanoue T, Sakamoto S, Itoh Y, Minami M, Yasui K, Sakamoto M, Nishioji K, Katagishi T, Nakagawa Y, Tada H, Sawa Y, Mizuno M, Kagawa K, Kashima K (1997). Side effects of high-dose interferon therapy for chronic hepatitis C. J Hepatol Gastroenterol Clin Biol.

